# 基于离子液体基低共熔溶剂的液液微萃取-超高效液相色谱-串联质谱法测定马尿中2种胰腺兴奋剂

**DOI:** 10.3724/SP.J.1123.2025.03019

**Published:** 2026-09-08

**Authors:** Qian CHEN, Zhichao MA, Yaonan LI

**Affiliations:** 武汉商学院，运动马检测及应用转化湖北省工程研究中心，湖北 武汉 430056; Wuhan Business University，Hubei Provincial Engineering Research Center of Racing Horse Detection and Application Transformation，Wuhan 430056，China

**Keywords:** 离子液体, 低共熔溶剂, 液液微萃取, 超高效液相色谱-串联质谱, 马尿, 醋磺己脲, 氯磺丙脲, ionic liquid （IL）, deep eutectic solvent （DES）, liquid-liquid microextraction, ultra-high performance liquid chromatography-tandem mass spectrometry （UHPLC-MS/MS）, equine urine, acetohexamide, chlorpropamide

## Abstract

利用离子液体（IL）作为氢键受体（HBA）合成了离子液体基低共熔溶剂（DES），配合涡旋辅助液-液微萃取（VALLME），结合超高效液相色谱-三重四极杆质谱法（UHPLC-MS/MS），开发了一种简单快速、绿色高效的预处理方法，用于提取和高灵敏检测马尿样中的胰腺兴奋剂。选择*N-*丁基甲基吗啉双三氟甲磺酰亚胺盐和五氟苯酚，以1∶4的物质的量之比合成IL-DES，测定了其共熔点，并对合成原料以及IL-DES进行了核磁分析和红外分析。优化后的萃取条件仅消耗100 μL萃取剂、1 mL尿样和6 min时间。随后采用外标法进行测定，醋磺己脲的线性范围为0.2~20 000 ng/mL，检出限（LOD）和定量限（LOQ）分别为0.1 ng/mL和0.2 ng/mL，氯磺丙脲的线性范围为0.5~20 000 ng/mL，决定系数（*r*^2^）均大于0.999，LOD和LOQ分别为0.2 ng/mL和0.5 ng/mL，基质效应的绝对值均小于20%，影响较弱，日内和日间相对标准偏差（RSD）≤6.6%，加标回收率为90.2%~110.4%，稳健性考察的RSD小于5.7%，残留影响均小于20%。与传统生物基质预处理方法相比，IL-DES-VALLME方法具有快速、简单、高效、低毒、低成本等优点，适用于马尿样品中胰腺兴奋剂的提取，在兴奋剂检测方面具有广阔的前景。

## Abstract (en)

Acetohexamide （AH） and chlorpropamide （CP） can stimulate pancreatic secretion of insulin and regulate water-salt balance， which may indirectly lead to an increase in adrenaline and potentially enhance the athletic performance of horses. For the protection of horse welfare and the consideration of fair competition， AH and CP were listed as pancreatic stimulants by the Federation Equestre Internationale （FEI）. A method for the detection of pancreatic stimulants in equestrian sports needs to be established. Before analyzing these drugs from biological samples such as urine， it is necessary to perform pre-treatment to separate and concentrate the target analytes. However， there are several disadvantages to using traditional pre-treatment methods， including toxicity and inefficiency. A rapid and environmentally friendly pre-treatment method utilizing ionic liquid-based deep eutectic solvent vortex-assisted liquid-liquid microextraction （IL-DES-VALLME） was developed. Coupled with ultra-high performance liquid chromatography-tandem mass spectrometry （UHPLC-MS/MS）， the approach enabled efficient extraction and sensitive quantification of AH and CP from equine urine. *N-*Butyl-*N-*methylmorpholinium bis（（trifluoromethyl）sulfonyl）imide （EMMPNTF_2_） was chosen as the hydrogen bond acceptor （HBA）， combined with pentafluorophenol （PFP） acting as the hydrogen bond donor （HBD）. A molar ratio of 1∶4 （HBA/HBD） was identified as the optimum composition of IL-DES. Differential scanning calorimetry （DSC） was utilized to measure the melting point of the synthesized IL-DES. Fourier-transform infrared （FT-IR） spectra and proton nuclear magnetic resonance （^1^H NMR） spectra were employed to characterize the structure of the IL-DES and investigate the hydrogen bonding interactions between its components. Under the optimum conditions， only 100 μL of IL-DES， 1 mL of equine urine， and 6 min were required. An external standard calibration method was utilized for quantification， and a linear relationship was achieved with a mass concentration range of 0.2-20 000 ng/mL for AH and 0.5-20 000 ng/mL for CP. The coefficients of determination （*r*^2^） were all greater than 0.999. The limits of detection （LOD） of the method based on a signal-to-noise ratio of 3 were 0.1 ng/mL for AH and 0.2 ng/mL for CP. The limits of quantification （LOQ） of the method based on a signal-to-noise ratio of 10 were 0.2 ng/mL for AH and 0.5 ng/mL for CP. The absolute values of matrix effects were all less than 20% （-8.9% for AH， -11.2% for CP）. Method accuracy， assessed through recovery studies at multiple concentrations across the linear range， yielded values between 90.2% and 110.4%. Precision， expressed as relative standard deviation （RSD）， was excellent， with intra-day RSDs≤6.6% and inter-day RSDs≤5.8%. The RSDs for robustness were less than 5.7%， and the average carry-over ratios were in the range of 11.4%-17.3%， indicating good method robustness and acceptable carry-over levels for the quantitation of AH and CP in equine urine. The greenness was assessed by the AGREEprep metric （score： 0.63）. Compared with traditional extraction methods， the IL-DES-VALLME method has the advantages of rapidity， simplicity， efficiency， low toxicity， and low cost. Additionally， the application of the proposed approach to real equine urine samples proved its practicability. These advantages make the method very appealing for doping control in equestrian sports.

醋磺己脲（acetohexamide， AH）和氯磺丙脲（chlorpropamide， CP）（结构式见图1）均隶属于第一代磺脲类降糖药，自20世纪50年代被研发出来并投入商业用途后，关于该类药物的副作用一直存在争议^［1］^。此类药物通过与胰岛B细胞膜上的特异性受体结合，导致三磷酸腺苷（adenosine triphosphate， ATP）敏感的钾离子通道关闭，诱发细胞膜去极化，引起细胞外钙离子内流，最终达到促进胰岛素分泌的作用，然而这种作用机制也会引发一系列反应^［2］^，如CP可不依赖抗利尿激素（antidiuretic hormone， ADH），直接增加内髓集合管（inner medullary collecting duct， IMCD）对水的通透性，产生抗利尿作用，进而扰乱动物体内水平衡，最终导致水潴留^［3］^。AH虽没有抗利尿特性，但可以通过增强肾脏中薄升支（thin ascending limb， tAL）和厚升支（thick ascending limb， TAL）对Na^+^、K^+^和Cl^-^的再吸收来增加游离水的量，从而引起低钾血症、低镁血症和低磷血症，某些情况下可促进肾上腺素分泌增加，进而提高动物的运动兴奋性^［4］^。鉴于上述两种药物可间接提高运动兴奋性，国际马术联合会（International Equestrian Federation， FEI）将AH和CP作为胰腺兴奋剂列入《国际马联马匹兴奋剂违禁物质清单》中^［5］^。考虑到马术运动赛事的公平性和马匹健康保护的福利性，有必要建立马术运动赛事中胰腺兴奋剂的检测方法。

**图1:**
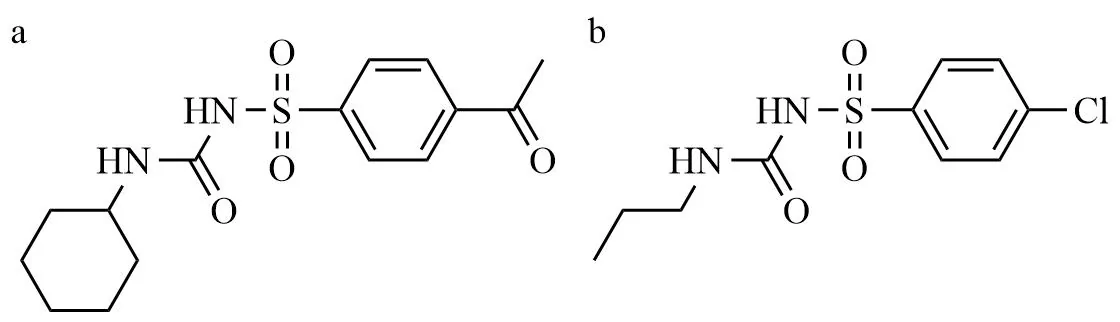
（a）AH和（b）CP的结构式

兴奋剂检测方法主要以血液、尿液等生物基质进行分析。截至目前，针对AH和CP的体液分析主要以人为研究对象，检测方法从早期的同位素稀释分析（isotopic dilution analysis， IDA）^［6］^发展到以色谱技术为核心的分析方法，包括胶束电动色谱法（MEKC）^［7］^、气相色谱法（GC）^［8-10］^、气相色谱-质谱法（GC-MS）^［11］^、高效液相色谱法（HPLC）^［12］^和液相色谱-电喷雾质谱法（LC-ESI-MS）^［13］^。Chua等^［14］^用HPLC来筛选检测马血浆样品中的CP，采用二氯甲烷萃取样品，并用GC-MS进行验证，检出限为3.5 ng/mL。经统计，上述人和马生物样品的色谱分析中，均采用甲苯、正己烷、二氯甲烷、乙酸乙酯、氯仿等有机溶剂进行目标物或衍生化目标物的液液萃取或液固萃取，这些前处理方法具有毒性大、时间长、萃取效率低的问题，因此亟待研发一种绿色高效的萃取剂来克服上述问题。

离子液体（ionic liquid， IL），亦称为“可设计性”溶剂，由于其不具有易燃性^［15］^，具有难挥发性^［16］^、热稳定性和化学稳定性^［17］^、高溶剂化能力^［18］^、高离子电导率^［19］^以及结构性能可调^［20］^等特性，在分离富集^［21］^、新能源^［22］^、有机合成^［23］^、制药^［24］^、核燃料后处理^［25］^、低共熔溶剂（deep eutectic solvent，DES）^［26］^等领域被研发应用。近年来，DES作为一种类似于离子液体的新型可持续绿色溶剂得到了进一步发展，其不仅具有IL的独特性能，而且黏度更低、合成成本更低、生物降解性更高^［27，28］^。DES通常由氢键受体（hydrogen bond acceptor，HBA）与氢键供体（hydrogen bond donor，HBD）混合而成，将IL盐或IL作为HBA合成的DES被称为离子液体基低共熔溶剂（IL-DES），主要用作高效溶解剂^［29］^、气体（CO_2_、SO_2_、NH_3_）捕获剂^［30-32］^、重金属离子去除剂^［33］^、石油采集剂^［34］^、催化剂介质^［35］^，充分展现出IL-DES优异的分离吸附性能。因此，本文采用14种离子液体与五氟苯酚（pentafluorophenol，PFP）设计合成不同的IL-DES，借助涡旋仪进行液液微萃取，研发出用于分离富集马尿中AH和CP的高效萃取剂，结合超高效液相色谱-三重四极杆质谱（UHPLC-MS/MS），遴选出最优萃取条件，并探讨了IL-DES的萃取机理，开发出适用于马术赛事中马尿胰腺兴奋剂的检测方法。

## 1 实验部分

### 1.1 仪器、试剂与材料

TSQ Altis Plus超高效液相色谱-三重四极杆质谱仪（Thermo Fisher Scientific，美国）；TGA/DSC 3+同步热分析仪（Mettler-Toledo，美国）；Nicolet iS10傅里叶变换红外吸收光谱仪（Thermo Fisher Scientific，美国）；Bruker AVANCE Ⅲ HD 600核磁共振波谱仪（Bruker，德国）；超纯水仪（Millipore，美国）；5427R台式高速冷冻离心机（Eppendorf，德国）；Cubis^®^ Ⅱ MCE2.7S-2CC*N-*F超微量天平（±0.1 μg，Sartorius，德国）；PB-30L pH计（Sartorius，德国）；ROCKER 2D digital涡旋混匀器（IKA，德国）。

胰腺兴奋剂标准品：AH（分析纯）、CP（分析纯）购自美国Sigma-Aldrich公司；*N-*丁基甲基吗啉双三氟甲磺酰亚胺盐（*N-*butyl-*N-*methylmorpholinium bis（（trifluoromethyl）sulfonyl）imide，EMMPNTF_2_，分析纯）购自上海成捷化学有限公司；PFP（分析纯）、氨水（ammonia solution，NH_4_OH，优级纯）购自上海阿拉丁生化科技股份有限公司；甲酸（formic acid，FA，质谱纯）、甲醇（methanol，MeOH，质谱纯）、乙腈（acetonitrile，ACN，质谱纯）购自美国Thermo Fisher Scientific公司。

### 1.2 标准溶液配制

精确称取两种胰腺兴奋剂标准品10.0 mg溶解于10 mL纯甲醇中，配制成1 mg/mL的单标储备溶液，再分别取AH单标储备溶液0.1 mL和CP单标储备溶液2 mL，混合于10 mL纯甲醇中，配制成混合标准储备溶液（AH：10 μg/mL，CP：200 μg/mL），保存在-80 ℃冰箱里。分析前，用现配的甲醇-水溶液（1∶9，体积比）将混合标准储备溶液稀释至所需浓度，制成混合标准工作溶液，于4 ℃冰箱短期保存备用。

### 1.3 IL-DES的制备

本实验采取直接电磁加热法合成IL-DES（DES 14）。首先，将PFP在常温干燥炉中干燥24 h，然后在10 mL透明玻璃瓶中快速称重2.192 1 g EMMPNTF_2_和3.681 2 g PFP，并在70 ℃下加热30 min，于室温（22 ℃）下放置过夜，最终获得约4 mL透明均质液体（可进行约50次萃取）。

### 1.4 萃取步骤

用10 μL甲酸将1 mL加标马尿液样品（AH：10 ng/mL，CP：200 ng/mL）的pH调节至5.00±0.01，用微量注射器将100 μL的DES 14萃取剂快速注射到样品中，涡旋2 min使混合液形成白色乳液，随后以12 000 r/min转速离心4 min，带有目标分析物的DES 14最后沉积于离心管底部，经0.22 μm尼龙膜过滤后转移至进样小瓶中，用于UHPLC-MS/MS检测。

### 1.5 UHPLC-MS/MS条件

UHPLC条件 Hypersil GOLD型C_18_色谱柱（100 mm×2.1 mm，3 μm，Thermo Fisher Scientific，美国）；流动相A是0.1%甲酸水溶液，B是纯甲醇。梯度洗脱程序如下：0~2 min，5%B；2~6 min，5%B~95%B；6~8 min，95%B；8~8.1 min，95%B~5%B；8.1~10 min，5%B。进样量10 μL。

质谱条件 电喷雾离子源，正离子模式，选择反应监测模式（selected reaction monitoring，SRM）；流速0.3 mL/min；电压3 500 V；离子传输管温度350 ℃；雾化器温度400 ℃，停留时间150 ms。

### 1.6 马尿样品收集

经武汉商学院马医院伦理委员会批准（批准编号：2023025），选择一匹12~14岁、无药物滥用史的公马进行实际马尿样品采集。早上7：00给马空腹喂服AH片剂500 mg、CP片剂200 mg，正常饮食饮水，收集自马服药后前两次排尿，作为阳性尿液样本。从10匹未服用相关药物的健康马身上采集空白尿液样本，在室温下振荡30 min，然后在12 000 r/min下离心10 min以去除杂质，取上清液与已活化的活性炭混匀，在室温下搅拌24 h，经真空抽滤后再用0.22 μm水系滤膜二次过滤，滤液在-80 ℃下冻存待用。分析前，用处理后的空白尿液样品将混合标准储备溶液稀释至所需浓度，制成加标尿液样品。

## 2 结果与讨论

### 2.1 质谱和色谱条件优化

质谱条件由自带软件TSQ Altis Tune 3.1优化而得，具体数据如表1所示，根据强度大小选择定量离子对，AH为*m*/*z* 324.9/199.4，CP为*m*/*z* 276.8/110.9。

**表1:** AH和CP的保留时间和质谱数据

Analyte	*t*_R_/min	Precursor （*m*/*z*）	Products （*m*/*z*）	CEs/V	RF lens/V
AH	6.92	324.9	199.4^*^， 243.8	17.76， 23.98	53
CP	6.83	276.8	110.9^*^， 175.1	31.33， 17.59	41

* Quantitative ion. CEs： collision energies； RF： radio frequency.

为使色谱条件达到最佳，以色谱峰面积为指标对流动相、柱温以及流速进行优化。首先对流动相中的有机相种类进行优化，结果显示甲醇的效果比乙腈更好，其次对水相中甲酸含量进行优化，依次使用纯水、0.05%甲酸水溶液、0.1%甲酸水溶液、0.2%甲酸水溶液与甲醇组合作为流动相进行梯度洗脱（同1.5节），在0.1%甲酸水溶液-甲醇流动相下，两种分析物的峰面积达到最高。随后对柱温进行优化，将柱温依次调至20、25、30、35、40、45 ℃，结果表明，随着柱温升高，两种分析物的峰面积和峰形无显著差异，考虑到柱压稳定性及常规操作习惯，选择30 ℃作为最佳柱温。最后对流速进行优化，保持梯度洗脱程序不变，依次将流速从0.1 mL/min调高至1.0 mL/min，每次增加0.1 mL/min，数据显示从0.3 mL/min开始，两种分析物的峰面积依次递减，因此最佳流速为0.3 mL/min。

### 2.2 萃取条件优化

#### 2.2.1 IL-DES种类筛选

萃取条件的优化以萃取回收率（ER）为指标进行衡量，ER计算公式见式（1）。

ER=*V*_a_*P*_a_/*V*_b_*P*_b_×100% （1）

其中*P*_a_是经IL-DES萃取后每个分析物的峰面积，*P*_b_是萃取前每个分析物的峰面积，*V*_a_是IL-DES的体积，*V*_b_是样品的体积。AH和CP的质量浓度分别固定在10 ng/mL和200 ng/mL，马尿样品体积固定在1 mL。

为制备IL-DES，选用14种常规的离子液体来作为HBA，固定PFP作为HBD，两者的物质的量之比都选用1∶2，考察它们在室温下的固液状态。如表2所示，当HBA为四丁基氯化膦六氟磷酸盐（B_4_PPF_6_）、四丁基膦双三氟甲磺酰亚胺盐（B_4_PNTF_2_）、*N*-丁基吡啶六氟磷酸盐（BpyPF_6_）、四丁基铵六氟磷酸盐（N_4444_PF_6_）、四丁基铵双三氟甲磺酰亚胺盐（N_4444_ NTF_2_）、*N*-丁基甲基吗啉六氟磷酸盐（EMMPPF_6_）时，所制备的IL-DES在室温下皆为固态，这可能是它们熔点较高的原因，其余8种IL-DES在室温下为液态，因此采用成功制备的8种IL-DES来进行最佳HBA的筛选。所制备的8种IL-DES从加标马尿样品中萃取AH和CP的能力如图2所示，DES 7、DES 9、DES 10和DES 14对AH的萃取率均处于较高水平，其中DES 14对CP的萃取率最高，因此，选择DES 14作为同时萃取两种分析物的最佳萃取剂。单独的离子液体EMMPNTF_2_作为萃取剂，其萃取率不足30%，远低于与PFP结合所形成的DES 14，进一步证明合成IL-DES的必要性。

**表2:** 14种IL-DES的组成成分、物质的量之比和室温下的状态

DES	HBA	HBD	Molar ratio	State at room temperature
DES 1	tetrabutylphosphonium hexafluorophosphate （B_4_PPF_6_）	PFP	1∶2	solid
DES 2	tetrabutylphosphonium bis（（trifluoromethyl）sulfonyl）imide （B_4_PNTF_2_）	PFP	1∶2	solid
DES 3	*N*-butyl-pyridinium hexafluorophosphate （BpyPF_6_）	PFP	1∶2	solid
DES 4	*N*-butyl-pyridinium bis（（trifluoromethyl）sulfonyl）imide （BpyNTF_2_）	PFP	1∶2	liquid
DES 5	tetrabutylammonium hexafluorophosphate （N_4444_PF_6_）	PFP	1∶2	solid
DES 6	tetrabutylammonium bis（（trifluoromethyl）sulfonyl）imide （N_4444_NTF_2_）	PFP	1∶2	solid
DES 7	*N*-butyl-*N*-methylpyrrolidinium hexafluorophosphate （PP_14_PF_6_）	PFP	1∶2	liquid
DES 8	*N*-butyl-*N*-methylpyrrolidinium bis（（trifluoromethyl）sulfonyl）imide （PP_14_NTF_2_）	PFP	1∶2	liquid
DES 9	*N-*butyl-*N-*methylpiperidinium hexafluorophosphate （P_14_PF_6_）	PFP	1∶2	liquid
DES 10	*N-*butyl-*N-*methylpiperidinium bis（（trifluoromethyl）sulfonyl）imide （P_14_NTF_2_）	PFP	1∶2	liquid
DES 11	1-butyl-3-methylimidazolium hexafluorophosphate （BMIMPF_6_）	PFP	1∶2	liquid
DES 12	1-butyl-3-methylimidazolium bis（（trifluoromethyl）sulfonyl）imide （BMIMNTF_2_）	PFP	1∶2	liquid
DES 13	*N*-butyl-*N*-methylmorpholinium hexafluorophosphate （EMMPPF_6_）	PFP	1∶2	solid
DES 14	*N*-butyl-*N*-methylmorpholinium bis（（trifluoromethyl）sulfonyl）imide （EMMPNTF_2_）	PFP	1∶2	liquid

DES： deep eutectic solvent； HBA： hydrogen bond acceptor； HBD： hydrogen bond donor； PFP： pentafluorophenol.

**图2:**
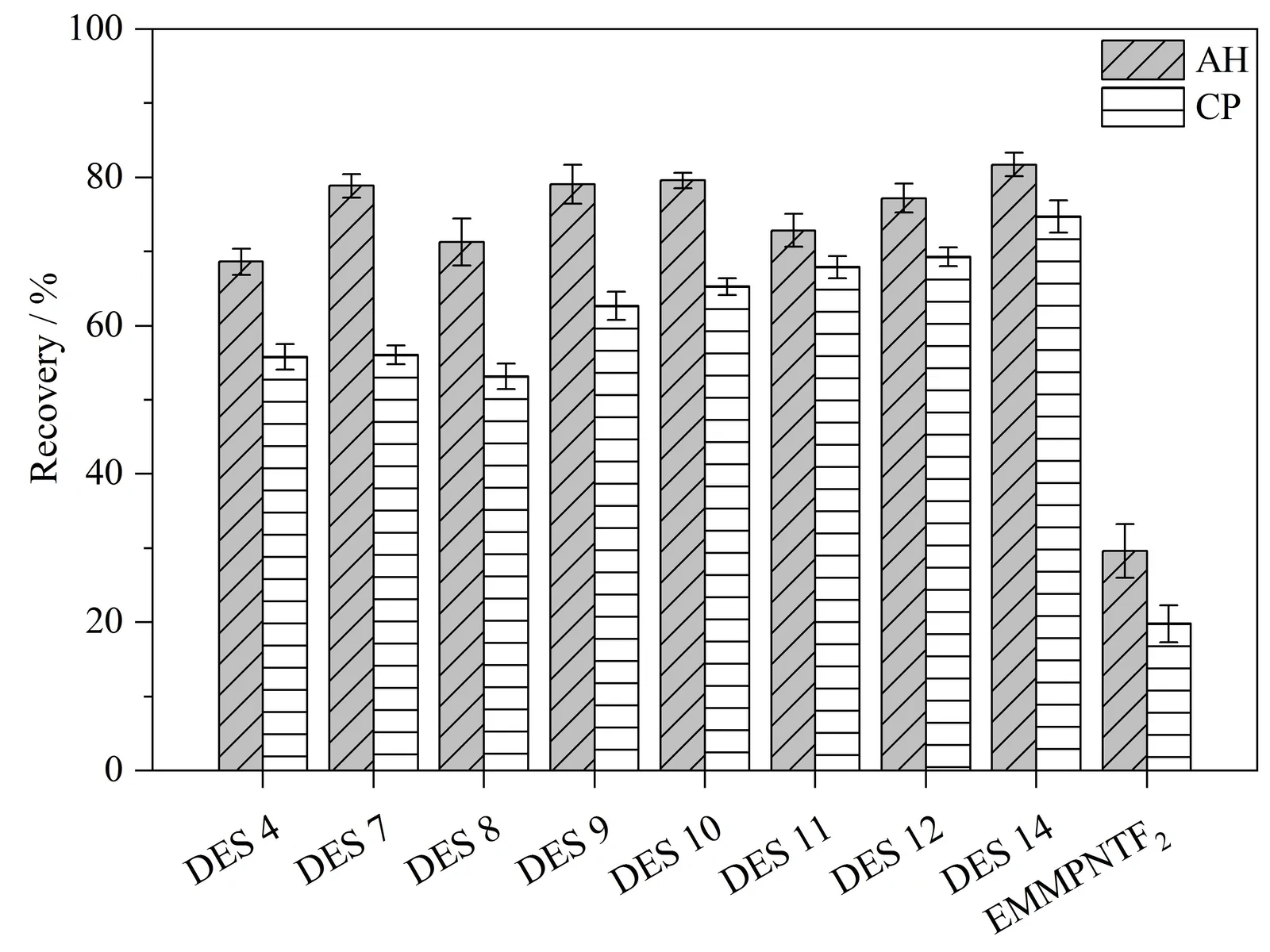
8种IL-DES和EMMPNTF_2_对马尿中AH和CP萃取率的影响（*n*=3）

#### 2.2.2 IL-DES中HBA与HBD的物质的量之比

对DES 14中HBA与HBD的物质的量之比进行优化。固定EMMPNTF_2_的量不变，增加PFP的量，使两者之间的物质的量之比依次为1∶1、1∶2、1∶3、1∶4、1∶5、1∶6、1∶7和1∶8，按照1.5节的流动相梯度条件分别对分析物的萃取率进行考查，如图3a所示，两种分析物的变化趋势一致，物质的量之比从1∶1变化至1∶4，萃取率相应升高，物质的量之比从1∶4变化至1∶8，萃取率先降低后趋于稳定，故EMMPNTF_2_与PFP的最佳物质的量之比为1∶4。

**图3:**
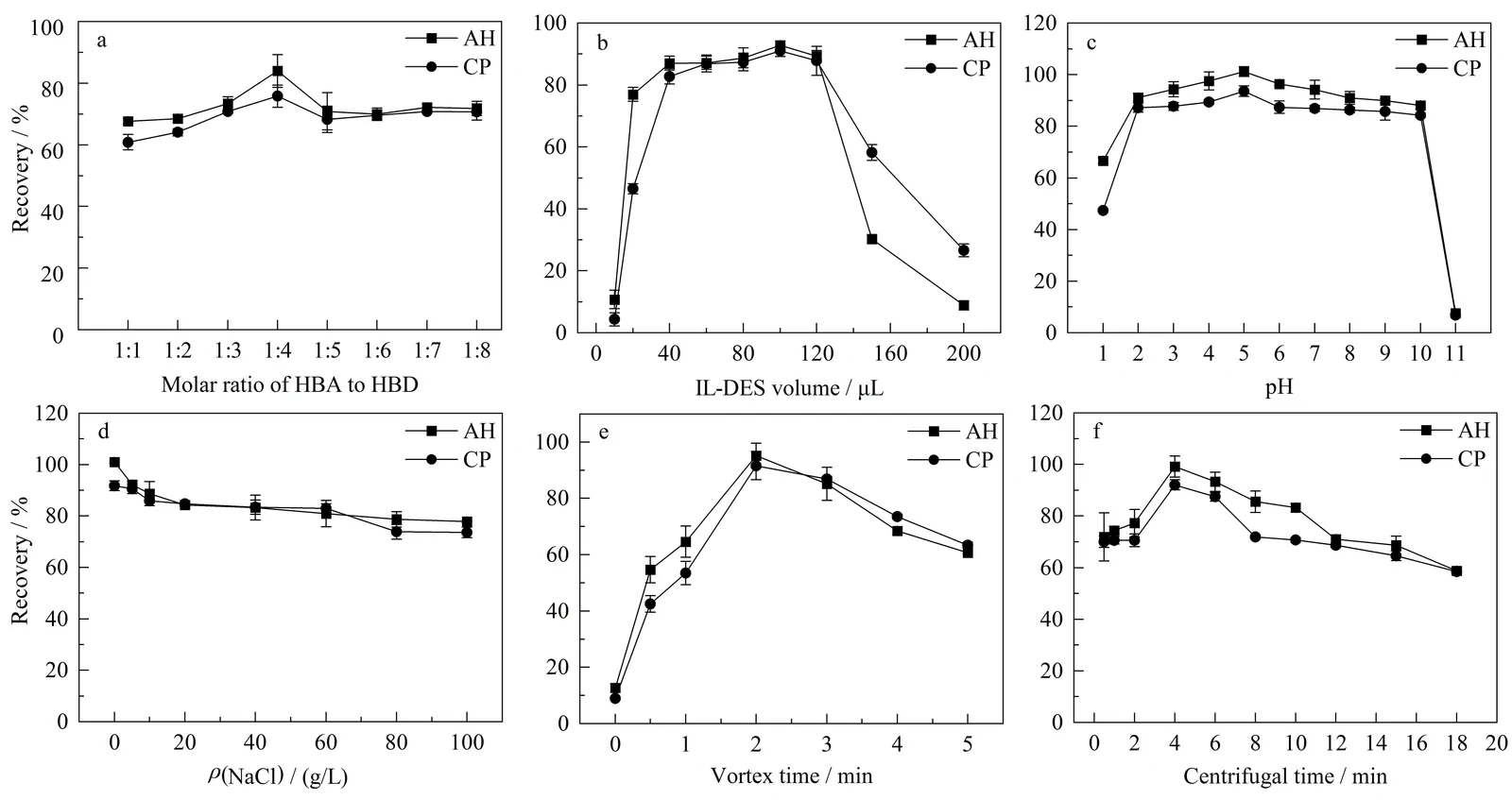
（a）HBA与HBD物质的量之比、（b）IL-DES用量、（c）pH、（d）NaCl质量浓度、（e）涡旋时间和（f）离心时间对马尿中AH和CP萃取率的影响（*n*=3）

#### 2.2.3 IL-DES用量

设置IL-DES用量为10、20、40、60、80、100、120、150、200 μL，依次考查对两种分析物的萃取能力。如图3b所示，当IL-DES用量低于40 μL时，萃取率处于较低水平；当IL-DES用量在40~120 μL范围内变化时，萃取率平缓上升，在100 μL时达到最高点后平缓下降；当IL-DES用量大于120 μL时，过量的萃取剂可能影响了涡旋分散效果，从而降低了传质效率，故IL-DES的最佳用量为100 μL。

#### 2.2.4 马尿样品的pH值

研究表明，通过控制pH来增加分析物在马尿样品中的中性分子数量，便于分析物转移至萃取剂，可获得较高的萃取回收率。因此考察了样品pH在1.00~11.00内IL-DES的萃取率，如图3c所示，当pH为1.00和11.00时，萃取率低，两种分析物主要以离子形式存在，对水相的亲和力大于对IL-DES的亲和力，表明强酸性和强碱性样品环境对IL-DES萃取能力有消极影响；当pH在2.00~10.00范围内，萃取率相对较高、变化较小，根据折线图变化趋势来看，当pH=5.00时，萃取率达到最高，因此最适pH为5.00。

#### 2.2.5 离子强度

为考察离子强度对IL-DES萃取AH和CP的影响，绘制了8种NaCl质量浓度下萃取率随离子强度变化趋势图。图3d表明，NaCl的添加对IL-DES的萃取率是抑制的，且随着NaCl浓度的升高，萃取率逐渐降低直至平缓。样品中存在的盐会减少萃取剂和尿样之间的密度差异，这不利于IL-DES分层，导致其难以分离，故在整个预处理过程中不添加NaCl。

#### 2.2.6 涡旋时间和离心时间

依次考察涡旋时间为0、0.5、1、2、3、4、5 min时的萃取率，以确定两种分析物的平衡时间。由图3e可知，涡旋能辅助萃取，涡旋2 min时两种分析物的萃取效率均达到顶峰，后随时间增长萃取效率降低，这是因为涡旋时间过长会破坏IL-DES与目标分析物的结合，不利于萃取。因此，2 min是IL-DES萃取AH和CP的最佳涡旋时间，此时涡旋与萃取能形成一种平衡状态。

为优化离心时间，选择10个时间点（0.5、1、2、4、6、8、10、12、15、18 min）来观察萃取效率的变化。其变化趋势（图3f）与涡旋时间一致，先增长再降低，在4 min时离心与萃取达到平衡，为最佳离心时间。

### 2.3 IL-DES表征

为探究IL-DES（DES 14，下同）的共熔点，对其进行示差扫描量热（DSC）分析，温度程序设置如下：从-90 ℃加热至-20 ℃，扫描速率为10 ℃/min。如图4所示，测得IL-DES的熔点为-80.1 ℃，远低于EMMPNTF_2_的熔点28.7 ℃和PFP的熔点35 ℃，表明两者之间产生相互作用形成了低共熔混合物。

**图4:**
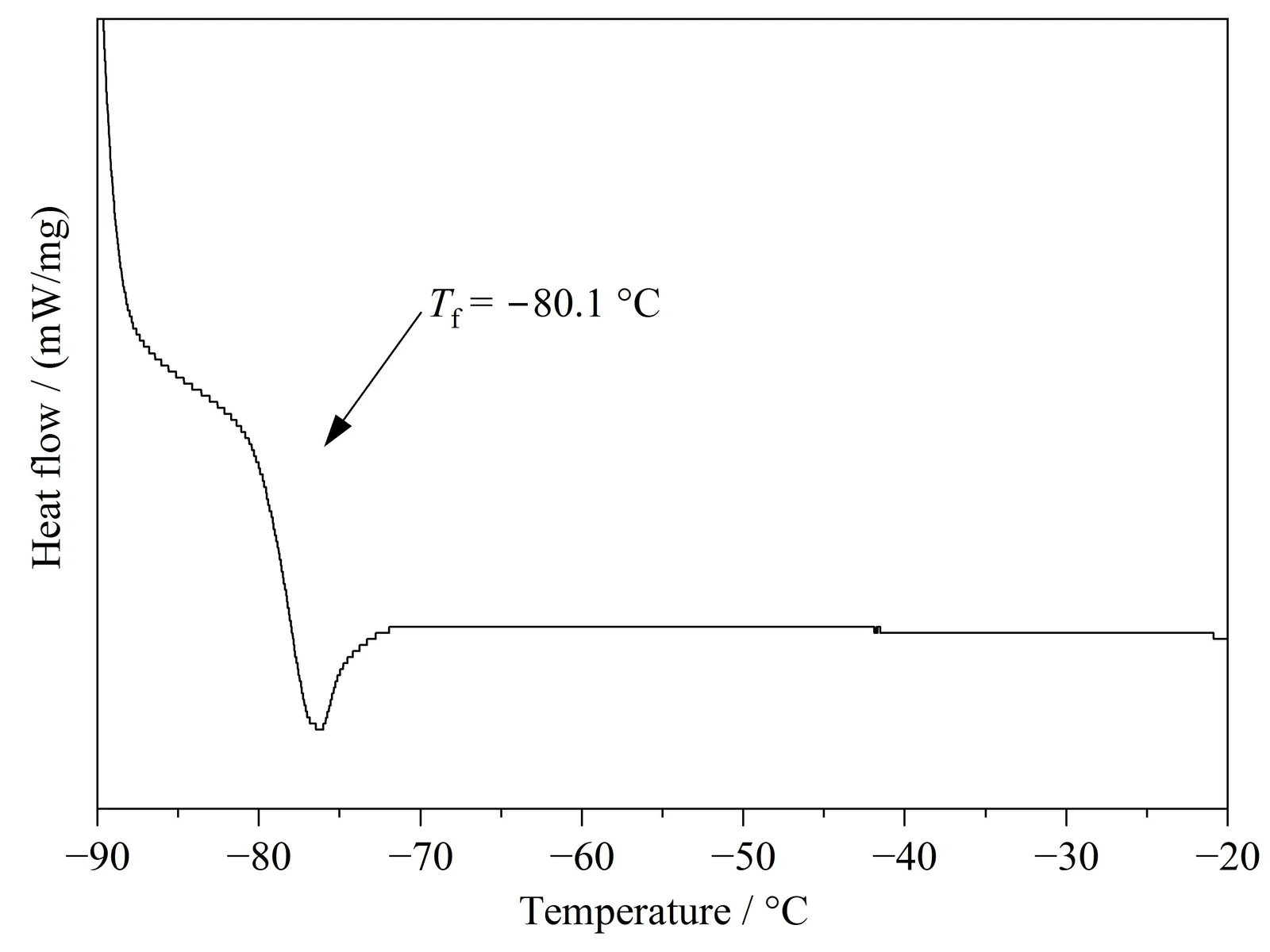
IL-DES（DES 14，下同）的差示扫描量热图

对PFP、EMMPNTF_2_和IL-DES进行核磁共振分析，如图5所示，EMMPNTF_2_的谱图中，氢信号主要归属于*N-*丁基-*N-*甲基吗啉阳离子，分别是0.97~1.02的N(CH_2_)_3_CH_3_，1.41~1.47的N(CH_2_)_2_CH_2_，1.76~1.82的NCH_2_CH_2_，3.22~3.24的NCH_3_，3.40~3.45的NCH_2_和3.46~3.52的CH_2_NCH_2_，4.03~4.09的CH_2_OCH_2_，与单体EMMPNTF_2_的谱图对比无明显变化。PFP中酚羟基的质子信号从合成前的5.69位移至合成后的5.85，且峰形加宽，表明PFP的-OH基团和EMMPNTF_2_之间形成了氢键，从而验证IL-DES的成功合成。

**图5:**
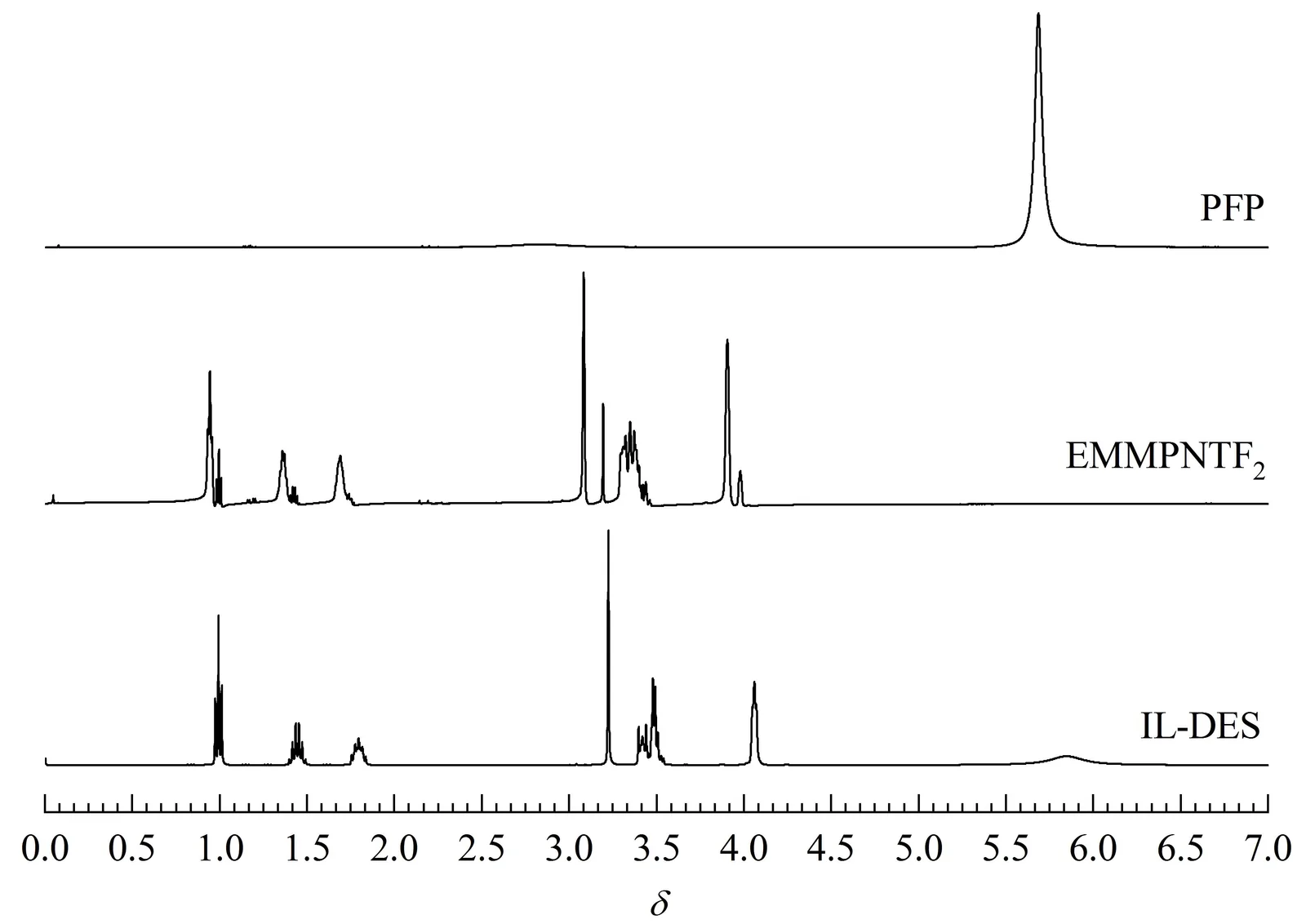
PFP，EMMPNTF_2_和IL-DES的核磁共振氢谱图

为进一步确定IL-DES的形成机理，对EMMPNTF_2_、PFP以及IL-DES萃取前后进行红外光谱分析，如图6所示，EMMPNTF_2_谱图中2 960 cm^-1^及2 870 cm^-1^处是吗啉环取代基上C-H的伸缩振动吸收峰，1 470 cm^-1^附近是C-H的弯曲振动吸收峰，1 340、1 130、1 170 cm^-1^是阴离子上S=O的伸缩振动峰，1 050 cm^-1^是吗啉环上C-O-C的伸缩振动吸收峰^［36］^。在PFP的谱图中3 580 cm^-1^处是苯酚的O-H伸缩振动吸收峰，1 510 cm^-1^处是苯环骨架上C=C的伸缩振动吸收峰，1 230 cm^-1^和1 310 cm^-1^是PFP上C-F的伸缩振动吸收峰。相比于EMMPNTF_2_和PFP的红外谱图，IL-DES谱图上3 580 cm^-1^处的O-H伸缩振动吸收峰消失，转而出现3 150~3 550 cm^-1^的O-H伸缩振动吸收带，意味着有多个分子间氢键的形成，同时S=O在1 340、1 130、1 170 cm^-1^处的伸缩振动吸收峰峰宽均变宽，其他峰位置和峰宽基本无变化，初步断定是EMMPNTF_2_的S=O和PFP的O-H形成了分子间氢键。经对比IL-DES萃取前后的红外谱图，各化学峰无明显变化，说明萃取剂与目标物没有发生化学反应，也体现出IL-DES的稳固性。

**图6:**
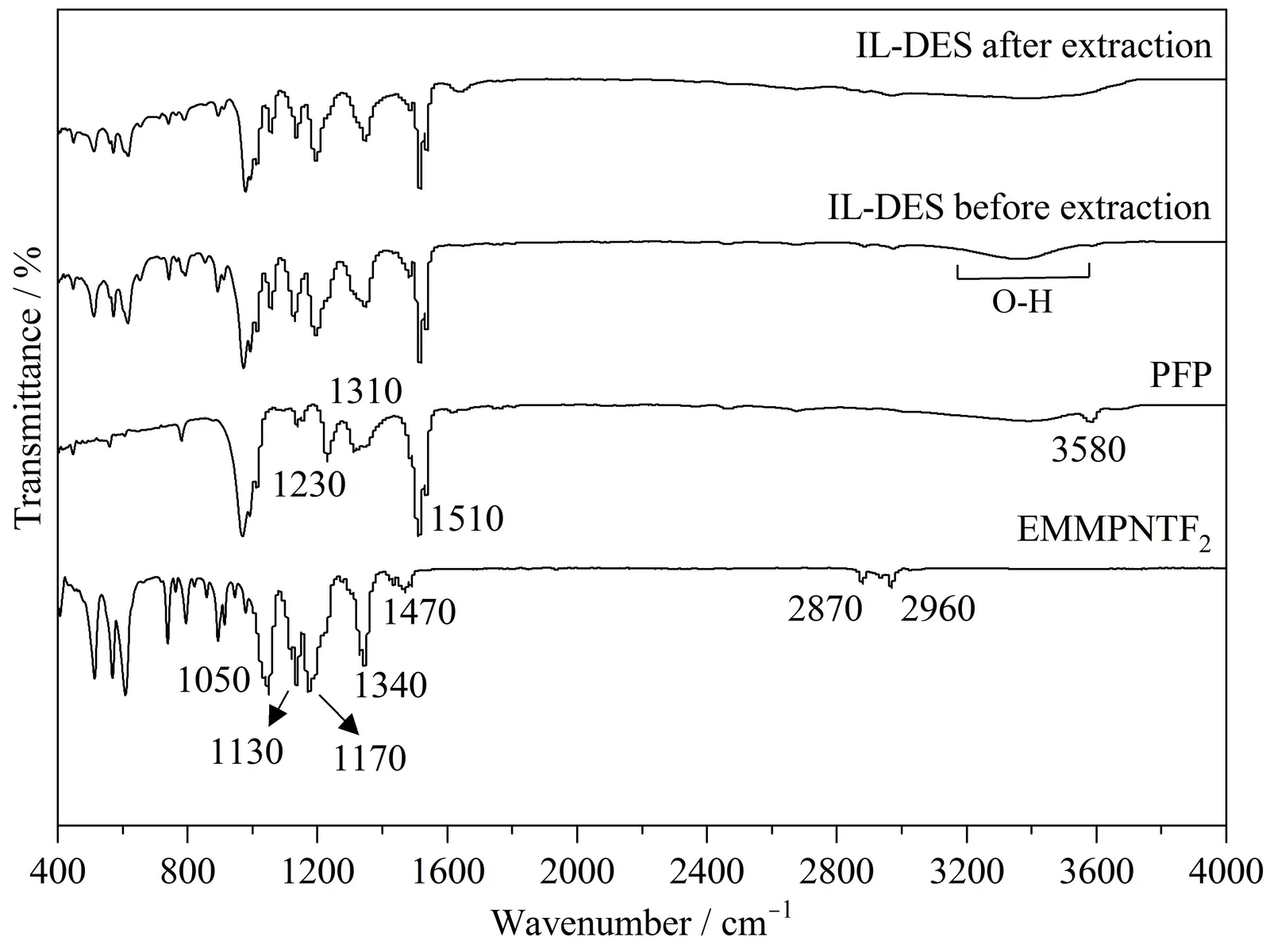
萃取后IL-DES、萃取前IL-DES、PFP和EMMPNTF_2_的红外光谱图

### 2.4 方法学考察

#### 2.4.1 线性范围、检出限和定量限

前处理流程优化完成后，进而实施定量分析方法的验证研究。在稀释的空白马尿液样品（稀释倍数为100）中加入一定质量浓度梯度的AH和CP（*n*=3），采用最小二乘法绘制两种分析物的峰面积（*y*，平均值）与质量浓度（*x*，平均值）的标准曲线，相关参数见表3。

**表3:** AH和CP的线性范围、回归方程、检出限和定量限（*n*=3）

Analyte	Linear range/（ng/mL）	Regression equation	Point number^a^	LOD/（ng/mL）	LOQ/（ng/mL）	*r*^2^
AH	0.2-20000	*y*=37.1*x*+994.9	16	0.1	0.2	0.9999
CP	0.5-20000	*y*=1005.7*x*+43854.5	15	0.2	0.5	0.9995

a. number of concentration points used for the calibration curves. *y*： peak area of the analyte； *x*： mass concentration of the analyte， ng/mL； LOD： limit of detection； LOQ： limit of quantification； *r*^2^： coefficient of determination.

#### 2.4.2 基质效应

考察马尿的基质效应（matrix effect，ME），采用ME=［（*S*_A_-*S*_B_）/*S*_B_］×100%公式进行计算，*S*_A_指采用IL-DES-VALLME方法萃取后的马尿配制的标准曲线斜率，*S*_B_指用流动相（0.1%甲酸水溶液-甲醇，5∶95，体积比）配制的标准曲线斜率。测得AH和CP的ME分别为-8.9%和-11.2%，绝对值均小于20%，表明马尿基质对两种分析物的信号抑制较弱，影响在可接受范围内。

#### 2.4.3 加标回收率和精密度

通过计算精密度（日内精密度和日间精密度）与回收率来验证方法的准确性。日内精密度（*n*=5）是通过在一天内检测不同加标浓度的马尿样品获得，而日间精密度（*n*=3）是通过连续3天分析相同浓度的马尿样品获得，回收率是标准曲线计算出的浓度与加标浓度的比值。表4显示，平均加标回收率范围在90.2%~110.4%的狭窄范围内，日内精密度≤5.8%，日间精密度≤6.6%，表明该方法具有较高的精确度与准确度。

**表4:** 不同水平下AH和CP在马尿中的加标回收率、日间和日内精密度

Analyte	Added/（ng/mL）	Intra-day （*n*=5）		Inter-day （*n*=3）	
		Recovery/%	RSD/%	Recovery/%	RSD/%
AH	0.2	96.1	1.6	93.0	5.5
	0.5	97.2	3.1	97.1	0.7
	1	97.6	4.6	99.0	6.6
	10	95.7	4.9	98.5	2.7
	100	97.4	2.9	97.7	3.3
	1000	97.0	1.2	96.8	1.8
	10000	107.4	3.7	106.4	2.1
	20000	108.4	5.2	103.7	2.6
CP	0.5	97.1	4.9	103.4	1.2
	1	101.1	5.8	105.1	3.5
	10	104.3	2.4	105.1	3.2
	100	90.4	0.8	90.2	1.0
	1000	105.4	1.9	106.1	2.4
	10000	110.4	1.6	109.7	2.1
	20000	90.6	0.6	104.8	1.4

#### 2.4.4 稳健性考察

稳健性研究是让两名实验员分别对10种不同的加标样品（质量浓度为10 ng/mL）进行每周1次（为期3周）的检测，来证明上述方法的稳健性。记录3周内的全部回收率数据，经计算AH和CP的RSD分别为2.2%和5.7%，这表明该时间段内的回收率没有显著变化，所开发的方法稳健性较高。

#### 2.4.5 残留考察

残留研究是先进样高浓度（定量上限）加标样品，紧接着进样空白样品，重复5次，计算残留峰面积与定量下限标准品峰面积的比值。AH和CP的平均比值分别为11.4%和17.3%，均小于20%，表明该方法的残留影响在可接受范围内。

### 2.5 前处理方法对比

对本文所提出的前处理方法与文献中的前处理方法进行梳理，围绕萃取剂类型和用量、耗时、线性范围、准确度、精确度和检出限等7个方面进行比较（表5）。发现在体液检测中对于AH的研究较少，CP偏多，且大多数围绕人体的血液和尿液进行方法开发，而本研究着眼于马尿基质进行前处理方法开发。经对比，IL-DES-VALLME方法中采用的IL-DES更加绿色环保，用量更少，仅需0.1 mL，耗时较短，线性范围更广，检出限更低，准确度和精确度的结果均在可接受范围内，能同时检测马尿中的AH和CP。

**表5:** 本方法和其他方法在生物样品中检测AH和CP的对比

Analyte	Sample	Extraction method	Extraction solvent	Extraction time/min	Detection technique	Linear range/（ng/mL）	Recovery/%	RSD/%	LOD/（ng/mL）	Ref.
CP	human plasma	LPE	*N-*hexane （5 mL）	—	GC	20000-100000	104-114	2.5-5.3	—	［^7^］
AH CP	human urine	SPE	methylene chloride （15 mL）	—	MEKC	—	—	—	50	［^8^］
AH	human plasma and urine	LPE	*N-*hexane （5 mL）	—	GC	5000-40000	plasma： 81-115 urine： 30-35	plasma： 3.0-11.5 urine： 8.0-16.0	—	［^9^］
CP	human plasma	LLE	chloroform （5 mL）	12	GC	—	94-100	—	—	［^10^］
CP	human plasma	LLE	ethyl acetate （6 mL）	>6	GC-MS	4000-44000	—	—	—	［^11^］
CP	human serum	LLE	toluene （1 mL）	>17	HPLC	2.5-300	78	<2.7	0.5	［^12^］
CP	human serum	SPE	ENVI-18 cartridge	>2	LC-ESI-MS	25-400	96.5	8.2	2-3	［^13^］
CP	horse plasma	LLE	dichloromethane （16 mL）	35	HPLC	0-300000	>95	1.2-3.6	3.5	［^14^］
AH CP	equine urine	IL-DES-VALLME	DES 14 （0.1 mL）	6	UHPLC-MS/MS	0.2-20000 0.5-20000	93.0-108.4 90.4-110.4	0.7-6.6 0.8-5.8	0.1 0.2	this work

LPE： liquid phase extraction； SPE： solid phase extraction； LLE： liquid-liquid extraction； IL-DES-VALLME： ionic liquid-based deep eutectic solvent vortex-assisted liquid-liquid microextraction； MEKC： micellar electrokinetic chromatography； GC-MS： gas chromatography-mass spectrometry； LC-ESI-MS： liquid chromatography-electrospray ionization-mass spectrometry； UHPLC-MS/MS： ultra-high performance liquid chromatography-tandem mass spectrometry. —： no data.

### 2.6 绿色度

为评估所开发方法的绿色环保性，使用在线程序AGREEprep进行打分。该程序的打分原理共包含十个独立的问题，根据每个问题输入相应的数据，最后汇总得到0~1区间的总分，其中1代表最高绿色度^［37］^。如图7所示，问题2、5、6和8的得分较高，表明该方法的有毒试剂用量少，样品量少，效率高且能耗低。问题3、4、7和10在材料可回收性、废物产生量、自动化程度、危害类型方面衡量，得分处于中间水平。因为采用离线方法制备样品和高精密度仪器检测样品，使得问题1和9得分较低。经计算得出最终的绿色度值为0.63，表明该方法具有一定绿色环保性。

**图7:**
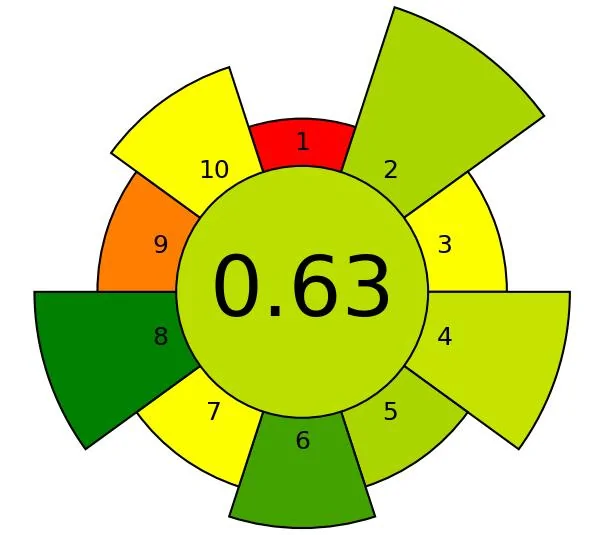
本方法的绿色度分布图

### 2.7 实际马尿样品检测

对所选取实验公马的阳性尿样进行分析，以证实所开发方法的可行性。阳性尿样的色谱图如图8所示，通过标准曲线计算得出AH和CP的质量浓度分别为369.1 ng/mL和286.6 ng/mL。此外，10份空白马尿样结果经检测均为未检出（阴性），这与马没有被喂食外源性AH和CP的事实一致。因此，该前处理方法和定量方法可用于胰腺兴奋剂的常规筛查。

**图8:**
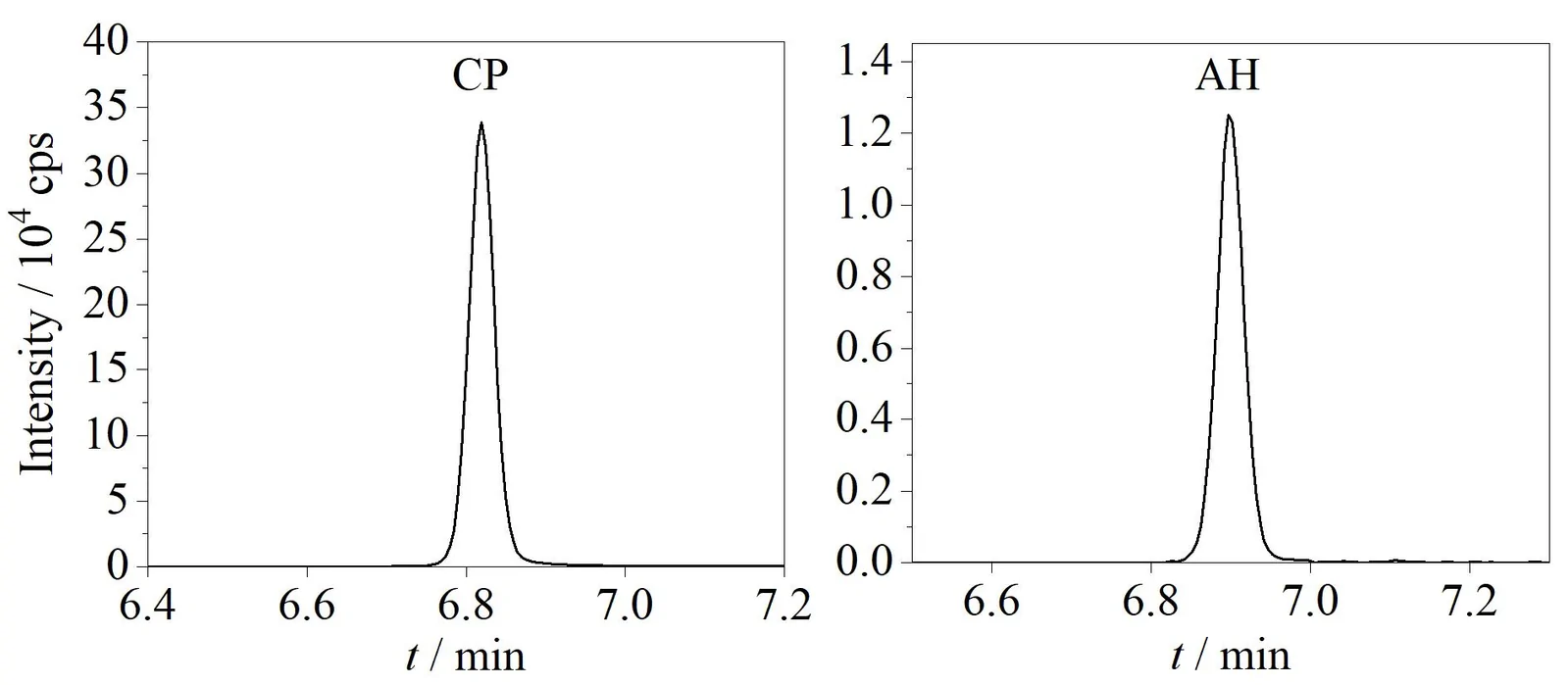
阳性马尿样品中CP和AH的色谱图

## 3 结论

开发了一种基于离子液体基低共熔溶剂的涡旋辅助液-液微萃取预处理方法，用于从马尿液中提取醋磺己脲和氯磺丙脲。该方法流程简单、提取效率高、成本低且环境友好，与超高效液相色谱-三重四极杆质谱结合，所建立的分析方法展现出可靠的准确度和精密度、优异的灵敏度和极佳的稳健性，并已成功应用于实际马尿样本分析。该方法在马术运动中醋磺己脲和氯磺丙脲类胰腺兴奋剂的检测方面具有广阔的应用前景。
